# XSuLT: a web server for structural annotation and representation of sequence-structure alignments

**DOI:** 10.1093/nar/gkx421

**Published:** 2017-05-16

**Authors:** Bernardo Ochoa-Montaño, Tom L. Blundell

**Affiliations:** Department of Biochemistry, University of Cambridge, Cambridge CB2 1GA, UK

## Abstract

The web server XSuLT, an enhanced version of the protein alignment annotation program JoY, formats a submitted multiple-sequence alignment using three-dimensional (3D) structural information in order to assist in the comparative analysis of protein evolution and in the optimization of alignments for comparative modelling and construct design. In addition to the features analysed by JoY, which include secondary structure, solvent accessibility and sidechain hydrogen bonds, XSuLT annotates each amino acid residue with residue depth, chain and ligand interactions, inter-residue contacts, sequence entropy, root mean square deviation and secondary structure and disorder prediction. It is also now integrated with built-in 3D visualization which interacts with the formatted alignment to facilitate inspection and understanding. Results can be downloaded as stand-alone HTML for the formatted alignment and as XML with the underlying annotation data. XSuLT is freely available at http://structure.bioc.cam.ac.uk/xsult/.

## INTRODUCTION

From the advent of macromolecular sequencing methods, the comparative analysis of biological sequences has been fundamental to the study of molecular evolution and function, as well as for practical applications in biotechnology and medicine. The alignment of sequences is a cornerstone technique in the development of many computational and statistical methods, providing a convenient and powerful representation of common features. However, it is often a challenging task and, in less than trivial cases, results may be dependent on context and interpretation, requiring experts to be able to visualize and analyse alignments efficiently. Numerous excellent tools have been developed for this purpose, notably Jalview (1), SeaView (2) and several others (3–8), both academic and commercial. While some programs provide functionality specific to certain sequence types, they are typically designed to support sequences generically, or tend to be more focused on genomic data. However, most proteins assume well defined three-dimensional (3D) structures, knowledge of which at the level of each residue provides a wealth of useful structural information that is usually not evident in typical sequence representations.

The program JoY (9) was developed almost 20 years ago to provide a bridge between sequence and structural information by implementing a typesetting format to represent a number of structural features of protein sequences in an alignment, with the goal of assisting in protein evolution studies, protein engineering and comparative modelling. It has since been routinely used in our own group and beyond (10–13). Nevertheless, despite the high information density achieved by the format, other relevant structural features, such as dynamic or interactive annotation, were not included in the original representation, often as it would have been impossible with the technology of the time.

Here we present the website XSuLT, an enhanced version of JoY that introduces additional functionality using modern web technologies. It is geared towards expanding the range of encoded features, improving user friendliness, and refining its utility for the process of comparative modelling and the development of constructs. Unlike the original JoY, which seeks to support a variety of output formats, for instance Postscript and Rich Text Format, XSuLT is fully focused on HTML output, which it generates by transforming an XML (Extensible Markup Language) file containing the annotated alignment using an XSLT (Extensible Stylesheet Language Transformations) template, from which the name of the program is derived. This way, all the data from the various sources are aggregated into a single, general and extensible file, which can be complexly formatted without specialized software in a wholly cross-platform manner, paving the way for customizing or incorporating additional features more easily in the future.

## MATERIALS AND METHODS

Like JoY, XSuLT is not designed to generate alignments of its own. Instead, it post-processes multiple sequence alignments (MSA) previously generated by other programs, using structural information from PDB files or predictions to annotate them. Due to this reliance, structural alignment programs like SALIGN (14), Expresso (15), POSA (16), MUSTANG (17), PDBeFold (18) or PROMALS3D (19) are recommended, but pure sequence alignment programs like MAFFT (20), Clustal-Omega (21) or T-Coffee (22) can also be used, particularly when the sequence similarity is above the twilight zone (23), i.e. in the range below 30–35% identity, where the probability of false positives increases sharply. The program can also operate on single structures to visualize their structural features.

**Figure 1.**
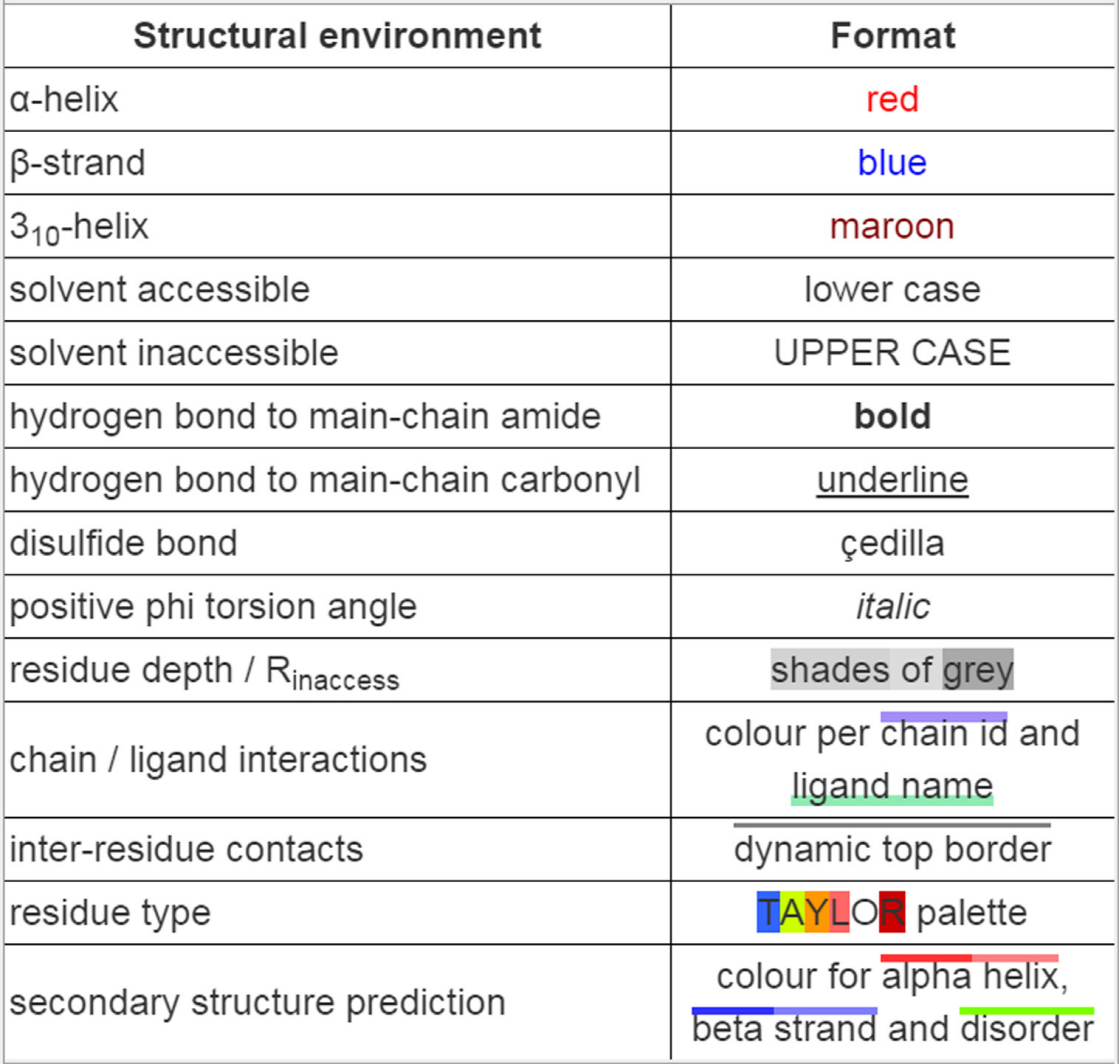
Key to XSuLT alignment formatting. Residue type and secondary structure prediction are only available for sequences without structural information.

XSuLT analyses each structure and sequence in the alignment, and the alignment as a whole, for the features described below. The alignment and all the annotations are stored as an XML file denominated as ‘XTEML’, the format of which is described in more detail in the Supplementary Materials. These data are transformed into a formatted HTML file displaying the annotations according to the key presented in Figure 1.

### Original JoY features

XSuLT relies on JoY for the analysis of the following core features, which are briefly outlined and described in more detail in the original publication (9):

**Residue solvent accessibility:** residues with a relative solvent-accessible surface area of <7% are defined as buried and displayed in uppercase letters (24).
**Secondary structure and main chain conformation:** three secondary structure classes are identified: α-helix, 3_10_ helix and β-strand, coloured in red, maroon and blue, respectively. Residues with positive mainchain φ angles (typically glycines) are also labelled using italics, due to their key role in important conformational features. In MSA, secondary structure consensus at a 70% threshold is also displayed on an additional line with a's on red background for alpha helices; b's on blue background for beta strands and 3's on orange background for 3_10_ helices.
**Hydrogen bonds:** residues with sidechain hydrogen bonds to main-chain amides are formatted in bold and those bonded to main-chain carbonyl are underlined.
**Physicochemical properties:** sequences without structural annotation are coloured according to the Taylor colour scheme (25), which assigns colour hues to certain physicochemical properties, such as hydrophobicity, aromaticity and polarity.

### Sequence-level features

The following newly added features are calculated for each individual structure or sequence, and generally displayed for each residue in them.

#### Residue depth and R_inaccess_

Like solvent accessibility (SA), depth is another metric related to the ‘buriedness’ of a residue, and has been found to be associated with characteristics like stability and conservation (26). Whereas SA refers to the surface of a residue exposed to the bulk solvent, depth relates to the distance of an atom or residue to that surface, allowing the quantitative discrimination between residues that lie just under the surface of the protein from those deep in its core. XSuLT can use the program EDTSurf (27) to calculate the average depth of each residue, and applies the value as a gradient of colour grey on the background of each residue, with darker shades indicating greater depth.

*R*_inaccess_ is a related but distinct metric introduced in the program Ghecom for the analysis of pockets in proteins (28). The program samples the surface of a protein with a series of spherical probes of a range of sizes to identify and characterize the shape of pockets. While protruding or flat regions of the surface can be reached by probes of any size, deeper cavities are only accessible to probes of smaller radius. *R*_inaccess_ stands for the minimum radius of inaccessible spherical probes, with smaller values generally correlating with deeper pockets. As with depth, XSuLT maps a grey shading of the background to this value, again with darker corresponding to deeper pockets.

Because of the overlap in formatting style, depth and *R*_inaccess_ cannot be represented simultaneously.

#### Chain and ligand interactions

The functions of most proteins involve interactions with other proteins, nucleic acids, carbohydrates or small molecules, many of them existing as part of multicomponent complexes. Interfaces and binding sites therefore constitute interesting features for comparison across related proteins. The structural interactomics database CREDO analyses and stores all interactions between molecules on the PDB (29). If XSuLT is used on a published PDB structure, it can query the database to annotate the residues in each sequence with information on whether they are in contact (i.e. within 5 Å) of another chain in the first biological assembly or with a small molecule ligand. Residues interacting with another chain are labelled with a coloured bar above it and those interacting with a ligand with a bar underneath it. The bars are coloured according to the identity of the chain or ligand; unfortunately, in cases interacting with more than one, only one can be displayed.

#### Inter-residue contacts

The shape of a protein can be fully represented by the distance pattern of its atoms, but even information about residues that are in contact with each other can assist in reconstructing the structure (30). The substitution of a residue during the evolution of a protein is constrained by its surrounding residues, which often leads to the co-evolution of those positions (31). Thanks to the large increase in biological sequence data, a variety of statistical and machine learning methods has been developed that are capable of predicting residue contacts for proteins with no structural information (32–35). Predicted contact maps have been successfully incorporated into *ab-initio* structure prediction programs to enhance their reach and accuracy (36–38).

XSuLT can analyse residue contacts in structures and display them on the alignment. Since contacts are relative to a specific position, this is done dynamically via interaction with the mouse cursor. Hovering with the mouse over a specific position will display a grey border above all residues within 6.0 Å of the aligned position and clicking on it will highlight them with a yellow background, to facilitate their identification.

#### Secondary structure and disorder prediction

Secondary structure is one of the most important structural features and several methods have been devized that are capable of predicting with a high degree of accuracy (39, 40). Regions of intrinsic disorder are increasingly recognized to be important structural and functional features in proteins (41) and the variety of methods (42–44) developed to predict them have become welcome tools to structural biologists, given the challenges they pose both in modelling and experimental structure determination. Such predictions can be useful to assess sequence-structure alignments, and XSuLT implements the use of PSIPRED 3.3 (39) and DISOPRED2 (45) for secondary structure and disorder prediction, respectively, in the analysis of non-structure sequences. Residues with a prediction confidence of at least 0.7 for helix or beta strand are labelled by a dark red or blue bar over the residue letter, respectively. Values between 0.7 and 0.3 are coloured in a lighter shade. Disorder predictions are presented in a light green colour when their confidence is at least eight. These are the same thresholds used by the utilities for the qualitative representation of their respective features.

In order to help assess generated models in the context of their alignment, it is also possible to enable this analysis on sequences with structures, provided that they are labelled as models in a PIR-formatted alignment by using the ‘structureM’ tag. Due to the time required to perform these predictions, when providing an alignment with multiple non-structure sequences, only the first one is analysed, if this option is selected.

#### Percentage sequence identity

When providing a mixed sequence-structure (or model-structure) alignment, as is often done for homology modelling, XSuLT calculates the percentage sequence identity (PID) of the first non-structure sequence to each of the structures, listing it at the end. The PID is a common metric to assess the degree of similarity of a sequence to its homologues or templates, but it was not previously shown on JoY.

### Alignment-level features

XSuLT also adds two further annotations calculated on a position level, taking into account all provided structures or sequences: Shannon entropy and Root Mean Square Deviation (RMSD).

#### Shannon entropy

The Shannon entropy is a concept from information theory related to the diversity or variability of states in a system. Applied to biological sequences in an alignment, it is useful as a measure of residue conservation (46). XSuLT implements a formula of normalized Shannon entropy using the gap treatment from Zhang (47):

\begin{equation*}\ S_i^{Sh} = \ \mathop \sum \limits_{a\ = \ 1}^{20} {f_{i,\ a}}{\log _n}{f_{i,\ a}} + {f_{i,\ {\rm gap}}}\end{equation*}

where *f_i,a_* is the relative frequency of amino acid *a* at alignment position *i, f_i_*,_gap_ is the relative frequency of gaps at the same position and *n* is the minimum of 20 and the number of sequences in the alignment. This formula yields values bounded between zero (when the position is totally conserved) and one (when all residues are different).

The calculated entropy is displayed on the formatted alignment as an additional row with values starting from * for fully conserved positions (i.e. *S* = 0) and over the 0–9 range for other entropy values over 0.1 intervals (i.e. 0 for 0 < *S* ≤ 0.1, 1 for 0.1 < *S* ≤ 0.2 and so on). Additionally, entropy is also available as a colour scheme in the 3D visualization, described on the following section, which allows to directly identify the regions on the structure that are most variable or conserved in terms of their sequence.

#### Root mean square deviation (RMSD)

Although structures are often presented as static, proteins exhibit considerable conformational flexibility. Consequently, even though the sequences of aligned proteins may be very similar or even identical, there might be significant differences in the spatial position of their atoms, with potential functional implications. Traditional alignment representations would be oblivious to them, raising the need for 3D visualization. However, while 3D superposition can highlight these differences, it also obscures essential information such as residue identity.

XSuLT addresses this conflict by annotating the alignment with the RMSD values of the aligned structures at each position. The values are obtained after superposing the structures using the program THESEUS (48). Its default setting of maximum likelihood superposition is used in order to downweigh the influence of flexible regions without needing to exclude them based on an arbitrary distance threshold.

The RMSD values of the Cα atoms at each aligned position are binned at a series of thresholds and presented with symbols corresponding to each of them: _ (RMSD < 2 Å), _=_ (<4 Å), 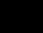 (<6 Å), 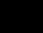 (<8 Å) and 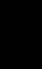 (≥8 Å). The symbols were chosen as a visually intuitive illustration of the closeness of the superposition that the RMSD represents. Thus, positions lying close in space are represented with a narrow underscore while those far apart are labelled with the full block. This makes it easy to recognize at first glance which regions of an alignment superpose more tightly and which ones are more flexible.

### 3D Visualization

A further enhancement of XSuLT with respect to JoY is its interactive integration with the light-weight 3D visualization plugin 3Dmol.js (49), providing a complementary visual context to the textual alignment. JavaScript is used to have the plugin react to mouse interactions with the alignment and assist in the analysis. Further details on its usage are provided in the following section below.

## WEB INTERFACE

XSuLT is freely available as a web server at http://structure.bioc.cam.ac.uk/xsult. Due to its reliance of modern technologies, only relatively recent browsers supporting HTML5 are supported, with Google Chrome recommended for best performance.

### Input

The program requires two types of input. First, an MSA (or individual sequence), which can be uploaded either as a file or pasted into the provided textbox. The server accepts two formats: FASTA and PIR/ALI. For the latter format, XSuLT conforms to the conventions used by MODELLER (50,51), described in detail on their documentation at http://salilab.org/modeller/manual/node496.html. The PIR format is recommended, as it allows specifying explicitly which sequences in the alignment are expected to have structural information. Moreover, it is also the only one to support labelling a sequence as a model, which enables the annotation of modelled (or experimental) structures with secondary structure predictions. As the fully MODELLER-compliant format can be cumbersome, XSuLT also supports a simplified version that only includes the labelling of each sequence as either a structure or a pure sequence. When using the FASTA format, any sequence for which no matching PDB is found, is automatically treated as a sequence. In either case, for performance reasons, the maximum number of sequences in a single alignment currently allowed is 25.

The structures of the sequences comprise the second required input. The only allowed format for this is PDB. There are two options available to provide the data, either by manually uploading the PDB files using the labelled button or by specifying the PDB and chain identifiers on the field provided.

It is essential that the sequence identifiers in the alignment match the PDB filenames and that the sequence in the alignment be identical to the residues in the PDB. If providing the PDB via its code and chain, it is worth verifying that the aligned sequence corresponds to the ATOM sequence of observed residues in the PDB, and not to the SEQRES sequence, which may contain residues not experimentally resolved, as this is a common source of error. The software will attempt to rectify automatically certain frequent issues with PDBs, but the user is advised to make sure their files are standards-compliant.

Once the job is submitted, the user is redirected to a page that will show its status and periodically refresh until the job is completed. Alternatively, if a problem occurs either due to an issue with the input data or the server, an error message with an explanation will be shown. In either case, the URL of the page can be saved and revisited later.

### Output

**Figure 2.**
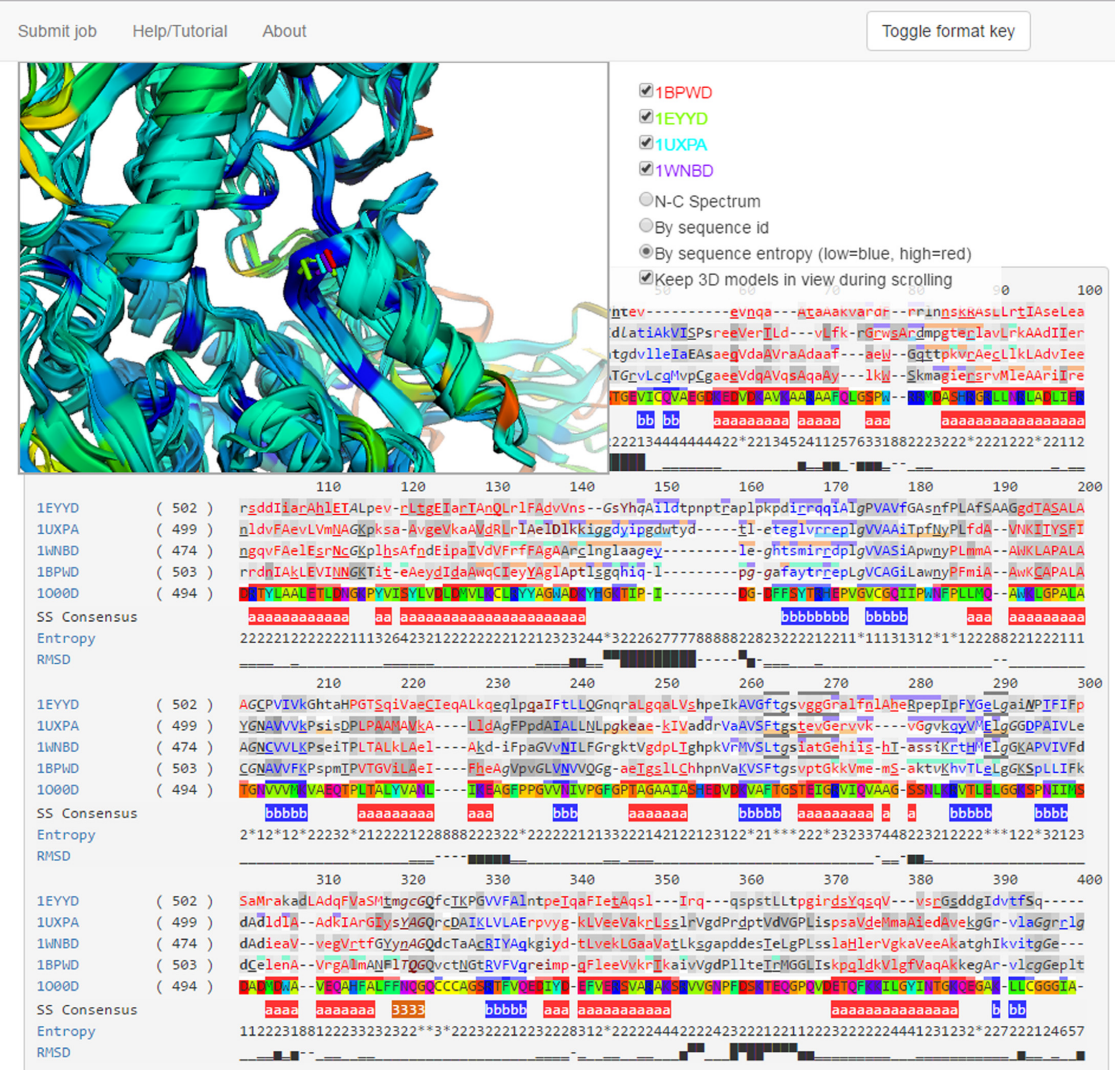
Example output of XSuLT. Results show the alignment of diverse aldehyde dehydrogenases (PDB IDs: 1EYY, 1UXP, 1WMN, 1BPW) with the sequence of the human one (PDB ID: 1O00, structure not shown). The residues shown correspond to position 260 on the alignment, where the mouse pointer (not shown) is hovering, which highlights neighbouring residues on the alignment with a grey top border. This position belongs to the conserved NADP binding site. The sequence 1UXP, which includes the ligand in its crystal structure, annotates the interacting residues with a pale orange bottom underlining.

The server output (Figure 2) consists of three sections: the 3D visualization of the aligned structures, the formatted alignment and the downloadable data. On the left hand side of the web page, recently submitted or opened jobs are listed and remembered for the duration of the browser session.

The 3D visualization box displays the superposition of the cartoon representation of all structures according to the provided alignment. Structures are initially coloured according to a rainbow spectrum, starting in red at the N-terminus and progressing to blue at the C-terminus, but two other colour schemes are also provided: each structure in a different colour and entropy spectrum, which colours residues according to their normalized Shannon entropy in a blue-green-red scale. Additionally, check boxes are provided to toggle the display of particular sequences and to keep the visualization box fixed in view during scrolling, in order to be able to interact with the end of long alignments.

The XSuLT formatted alignment constitutes the primary output of the program. It shows the provided alignment annotated according to the analyses described previously using the format shown on Figure 1 and detailed in the ‘Materials and Methods’ section. The ‘Toggle format key’ button at the top of the page can be used at any time to display the formatting key and hovering with the mouse pointer over any position will show details about its annotated features. Sequences with an associated PDB file are automatically linked to their entry on the Protein Data Bank website.

Hovering with the mouse over an aligned position will also display the stick representation of the sidechains on the 3D visualization box, and clicking on it will zoom and centre the view on it. This can be useful to identify easily any misaligned residues and to optimize the structural equivalence of ambiguous positions.

At the end of the results page, several generated files are made available for download:

The PIR formatted alignment, including any edits performed to make it fully compatible with MODELLER.
A compressed ZIP file containing the superposed PDB files.
A stand-alone HTML file of the formatted alignment for reference and off-site viewing. However, due to the reliance on a number of JavaScript libraries (namely LESS, jQuery and 3Dmol.js), it is still necessary to be online to view them. Due to technical limitations, this HTML file requires the user to load the PDB files manually for the 3D visualization using the button provided. Additionally, the file includes the ability to extract the alignment in FASTA format using the button at the bottom. A trimmed down version without 3D and buttons more suitable for printing is also available.
The XTEML file containing the alignment and all the generated annotation data, which the user can process if they are interested in any of the raw data.

Generated results are kept on the server as long as storage capacity allows, for a minimum of one month.

## DISCUSSION AND CONCLUSION

The features analysed and mapped by JoY and now XSuLT onto one-letter amino acid alignments allow for the rapid identification of structural features that are likely to be important to the fold or function. With the addition of the integrated 3D visualization, this ability is further enhanced.

The example presented on Figure 2 shows the sequence-structure alignment of five diverse NADP+ dependent aldehyde dehydrogenases (PDB IDs: 1EYY (52), 1UXP (53), 1WNB (54), 1BPW (55)) with the sequence of the human one (PDB ID: 1O00 (56)), whose structure was not provided for illustration purposes. Even without displaying any ligands on the 3D visualization, it is easy to identify the binding site residues for NADP both on the alignment, via the annotation from CREDO around positions 260–270, and on the 3D box with sequence entropy view, which shows the pocket of low entropy in blue, indicating a high conservation of the residues involved. The sequence for 1UXP also shows a second, less conserved, ligand binding site for AMP around positions 145 and 170, which can be seen to be allosteric either directly on the 3D representation or through the dynamic contacts formatting that show it does not lie in the vicinity of the catalytic site. Likewise, XSuLT also shows that the sequences are part of oligomeric complexes and facilitates the identification of all interfacial residues spread over the sequences.

Our group routinely uses JoY and XSuLT for the preparation and analysis of new structures (10–13) and has been integrated into a number of databases (57–59). XSuLT is integral to our upcoming database TOCCATA (http://structure.bioc.cam.ac.uk/toccata, manuscript in preparation), which classifies chains and domains into consensus categories from SCOP (60) and CATH (61) families for use in the remote homology detection program FUGUE and for homology modelling.

The new architecture based on XML and XSLT simplifies the process of adding annotations and customizing the output and we plan to take advantage of this to integrate more sources of data in the future.

## Supplementary Material

Supplementary Data

## References

[1] Waterhouse A.M., Procter J.B., Martin D.M.A., Clamp M., Barton G.J. Jalview Version 2–a multiple sequence alignment editor and analysis workbench. Bioinformatics. 2009; 25:1189–1191. 19151095 10.1093/bioinformatics/btp033 PMC2672624

[2] Gouy M., Guindon S., Gascuel O. SeaView version 4: a multiplatform graphical user interface for sequence alignment and phylogenetic tree building. Mol. Biol. Evol. 2010; 27:221–224. 19854763 10.1093/molbev/msp259

[3] Caffrey D.R., Dana P.H., Mathur V., Ocano M., Hong E.-J., Wang Y.E., Somaroo S., Caffrey B.E., Potluri S., Huang E.S. PFAAT version 2.0: a tool for editing, annotating, and analyzing multiple sequence alignments. BMC Bioinformatics. 2007; 8:381. 17931421 10.1186/1471-2105-8-381 PMC2092438

[4] Okonechnikov K., Golosova O., Fursov M. UGENE team Unipro UGENE: a unified bioinformatics toolkit. Bioinformatics. 2012; 28:1166–1167. 22368248 10.1093/bioinformatics/bts091

[5] Gille C., Fähling M., Weyand B., Wieland T., Gille A. Alignment-annotator web server: rendering and annotating sequence alignments. Nucleic Acids Res. 2014; 42:W3–W6. 24813445 10.1093/nar/gku400 PMC4086088

[6] Yachdav G., Wilzbach S., Rauscher B., Sheridan R., Sillitoe I., Procter J., Lewis S.E., Rost B., Goldberg T. MSAViewer: interactive JavaScript visualization of multiple sequence alignments. Bioinformatics. 2016; 32:3501–3503. 27412096 10.1093/bioinformatics/btw474 PMC5181560

[7] Jehl P., Manguy J., Shields D.C., Higgins D.G., Davey N.E. ProViz-a web-based visualization tool to investigate the functional and evolutionary features of protein sequences. Nucleic Acids Res. 2016; 44:W11–W15. 27085803 10.1093/nar/gkw265 PMC4987877

[8] Veidenberg A., Medlar A., Löytynoja A. Wasabi: an integrated platform for evolutionary sequence analysis and data visualization. Mol. Biol. Evol. 2016; 33:1126–1130. 26635364 10.1093/molbev/msv333

[9] Mizuguchi K., Deane C.M., Blundell T.L., Johnson M.S., Overington J.P. JOY: protein sequence-structure representation and analysis. Bioinformatics. 1998; 14:617–623. 9730927 10.1093/bioinformatics/14.7.617

[10] Nookala R.K., Langemeyer L., Pacitto A., Ochoa-Montaño B., Donaldson J.C., Blaszczyk B.K., Chirgadze D.Y., Barr F.A., Bazan J.F., Blundell T.L. Crystal structure of folliculin reveals a hidDENN function in genetically inherited renal cancer. Open Biol. 2012; 2:120071. 22977732 10.1098/rsob.120071 PMC3438538

[11] Ochi T., Wu Q., Chirgadze D.Y., Grossmann J.G., Bolanos-Garcia V.M., Blundell T.L. Structural insights into the role of domain flexibility in human DNA ligase IV. Structure. 2012; 20:1212–1222. 22658747 10.1016/j.str.2012.04.012 PMC3391681

[12] Ochi T., Blackford A.N., Coates J., Jhujh S., Mehmood S., Tamura N., Travers J., Wu Q., Draviam V.M., Robinson C.V. PAXX, a paralog of XRCC4 and XLF, interacts with Ku to promote DNA double-strand break repair. Science. 2015; 347:185–188. 25574025 10.1126/science.1261971 PMC4338599

[13] Sibanda B.L., Chirgadze D.Y., Ascher D.B., Blundell T.L. DNA-PKcs structure suggests an allosteric mechanism modulating DNA double-strand break repair. Science. 2017; 355:520–524. 28154079 10.1126/science.aak9654

[14] Braberg H., Webb B.M., Tjioe E., Pieper U., Sali A., Madhusudhan M.S. SALIGN: a web server for alignment of multiple protein sequences and structures. Bioinformatics. 2012; 28:2072–2073. 22618536 10.1093/bioinformatics/bts302 PMC3400954

[15] Armougom F., Moretti S., Poirot O., Audic S., Dumas P., Schaeli B., Keduas V., Notredame C. Expresso: automatic incorporation of structural information in multiple sequence alignments using 3D-Coffee. Nucleic Acids Res. 2006; 34:W604–W608. 16845081 10.1093/nar/gkl092 PMC1538866

[16] Li Z., Natarajan P., Ye Y., Hrabe T., Godzik A. POSA: a user-driven, interactive multiple protein structure alignment server. Nucleic Acids Res. 2014; 42:W240–W245. 24838569 10.1093/nar/gku394 PMC4086100

[17] Konagurthu A.S., Whisstock J.C., Stuckey P.J., Lesk A.M. MUSTANG: a multiple structural alignment algorithm. Proteins. 2006; 64:559–574. 16736488 10.1002/prot.20921

[18] Krissinel E., Henrick K. Secondary-structure matching (SSM), a new tool for fast protein structure alignment in three dimensions. Acta Crystallogr. D Biol. Crystallogr. 2004; 60:2256–2268. 15572779 10.1107/S0907444904026460

[19] Pei J., Tang M., Grishin N.V. PROMALS3D web server for accurate multiple protein sequence and structure alignments. Nucleic Acids Res. 2008; 36:W30–W34. 18503087 10.1093/nar/gkn322 PMC2447800

[20] Katoh K., Standley D.M. MAFFT multiple sequence alignment software version 7: improvements in performance and usability. Mol. Biol. Evol. 2013; 30:772–780. 23329690 10.1093/molbev/mst010 PMC3603318

[21] Sievers F., Wilm A., Dineen D., Gibson T.J., Karplus K., Li W., Lopez R., McWilliam H., Remmert M., Söding J. Fast, scalable generation of high-quality protein multiple sequence alignments using Clustal Omega. Mol. Syst. Biol. 2011; 7:539. 21988835 10.1038/msb.2011.75 PMC3261699

[22] Notredame C., Higgins D.G., Heringa J. T-Coffee: a novel method for fast and accurate multiple sequence alignment. J. Mol. Biol. 2000; 302:205–217. 10964570 10.1006/jmbi.2000.4042

[23] Rost B. Twilight zone of protein sequence alignments. Protein Eng. 1999; 12:85–94. 10195279 10.1093/protein/12.2.85

[24] Hubbard T.J., Blundell T.L. Comparison of solvent-inaccessible cores of homologous proteins: definitions useful for protein modelling. Protein Eng. 1987; 1:159–171. 3507702 10.1093/protein/1.3.159

[25] Taylor W.R. Residual colours: a proposal for aminochromography. Protein Eng. Des. Sel. 1997; 10:743–746. 10.1093/protein/10.7.743 9342138

[26] Chakravarty S., Varadarajan R. Residue depth: a novel parameter for the analysis of protein structure and stability. Structure. 1999; 7:723–732. 10425675 10.1016/s0969-2126(99)80097-5

[27] Xu D., Li H., Zhang Y. Protein depth calculation and the use for improving accuracy of protein fold recognition. J. Comput. Biol. J. Comput. Mol. Cell Biol. 2013; 20:805–816. 10.1089/cmb.2013.0071 PMC3837563 23992298

[28] Kawabata T. Detection of multiscale pockets on protein surfaces using mathematical morphology. Proteins. 2010; 78:1195–1211. 19938154 10.1002/prot.22639

[29] Schreyer A.M., Blundell T.L. CREDO: a structural interactomics database for drug discovery. Database (Oxford). 2013; 2013:bat049. 23868908 10.1093/database/bat049 PMC3715132

[30] Vendruscolo M., Kussell E., Domany E. Recovery of protein structure from contact maps. Fold Des. 1997; 2:295–306. 9377713 10.1016/S1359-0278(97)00041-2

[31] Göbel U., Sander C., Schneider R., Valencia A. Correlated mutations and residue contacts in proteins. Proteins. 1994; 18:309–317. 8208723 10.1002/prot.340180402

[32] Seemayer S., Gruber M., Söding J. CCMpred–fast and precise prediction of protein residue-residue contacts from correlated mutations. Bioinformatics. 2014; 30:3128–3130. 25064567 10.1093/bioinformatics/btu500 PMC4201158

[33] Kamisetty H., Ovchinnikov S., Baker D. Assessing the utility of coevolution-based residue-residue contact predictions in a sequence- and structure-rich era. Proc. Natl. Acad. Sci. U.S.A. 2013; 110:15674–15679. 24009338 10.1073/pnas.1314045110 PMC3785744

[34] Jones D.T., Singh T., Kosciolek T., Tetchner S. MetaPSICOV: combining coevolution methods for accurate prediction of contacts and long range hydrogen bonding in proteins. Bioinformatics. 2015; 31:999–1006. 25431331 10.1093/bioinformatics/btu791 PMC4382908

[35] Wang S., Sun S., Li Z., Zhang R., Xu J. Accurate de novo prediction of protein contact map by ultra-deep learning model. PLoS Comput. Biol. 2017; 13:e1005324. 28056090 10.1371/journal.pcbi.1005324 PMC5249242

[36] Adhikari B., Bhattacharya D., Cao R., Cheng J. CONFOLD: residue-residue contact-guided ab initio protein folding. Proteins. 2015; 83:1436–1449. 25974172 10.1002/prot.24829 PMC4509844

[37] Kosciolek T., Jones D.T. De novo structure prediction of globular proteins aided by sequence variation-derived contacts. PLoS One. 2014; 9:e92197. 24637808 10.1371/journal.pone.0092197 PMC3956894

[38] Marks D.S., Colwell L.J., Sheridan R., Hopf T.A., Pagnani A., Zecchina R., Sander C. Protein 3D structure computed from evolutionary sequence variation. PLoS One. 2011; 6:e28766. 22163331 10.1371/journal.pone.0028766 PMC3233603

[39] Jones D.T. Protein secondary structure prediction based on position-specific scoring matrices. J. Mol. Biol. 1999; 292:195–202. 10493868 10.1006/jmbi.1999.3091

[40] Cuff J.A., Barton G.J. Application of multiple sequence alignment profiles to improve protein secondary structure prediction. Proteins. 2000; 40:502–511. 10861942 10.1002/1097-0134(20000815)40:3<502::aid-prot170>3.0.co;2-q

[41] Dunker A.K., Brown C.J., Lawson J.D., Iakoucheva L.M., Obradović Z. Intrinsic disorder and protein function. Biochemistry (Mosc.). 2002; 41:6573–6582. 10.1021/bi012159+ 12022860

[42] Deng X., Eickholt J., Cheng J. A comprehensive overview of computational protein disorder prediction methods. Mol. Biosyst. 2012; 8:114–121. 21874190 10.1039/c1mb05207a PMC3633217

[43] Atkins J.D., Boateng S.Y., Sorensen T., McGuffin L.J. Disorder prediction methods, their applicability to different protein targets and their usefulness for guiding experimental studies. Int. J. Mol. Sci. 2015; 16:19040–19054. 26287166 10.3390/ijms160819040 PMC4581285

[44] Li J., Feng Y., Wang X., Li J., Liu W., Rong L., Bao J. An overview of predictors for intrinsically disordered proteins over 2010-2014. Int. J. Mol. Sci. 2015; 16:23446–23462. 26426014 10.3390/ijms161023446 PMC4632708

[45] Ward J.J., Sodhi J.S., McGuffin L.J., Buxton B.F., Jones D.T. Prediction and functional analysis of native disorder in proteins from the three kingdoms of life. J. Mol. Biol. 2004; 337:635–645. 15019783 10.1016/j.jmb.2004.02.002

[46] Valdar W.S.J. Scoring residue conservation. Proteins. 2002; 48:227–241. 12112692 10.1002/prot.10146

[47] Zhang S.-W., Zhang Y.-L., Pan Q., Cheng Y.-M., Chou K.-C. Estimating residue evolutionary conservation by introducing von Neumann entropy and a novel gap-treating approach. Amino Acids. 2008; 35:495–501. 17710364 10.1007/s00726-007-0586-0 PMC7088136

[48] Theobald D.L., Wuttke D.S. THESEUS: maximum likelihood superpositioning and analysis of macromolecular structures. Bioinformatics. 2006; 22:2171–2172. 16777907 10.1093/bioinformatics/btl332 PMC2584349

[49] Rego N., Koes D. 3Dmol.js: molecular visualization with WebGL. Bioinformatics. 2015; 31:1322–1324. 25505090 10.1093/bioinformatics/btu829 PMC4393526

[50] Sali A., Blundell T.L. Comparative protein modelling by satisfaction of spatial restraints. J. Mol. Biol. 1993; 234:779–815. 8254673 10.1006/jmbi.1993.1626

[51] Webb B., Sali A. Comparative protein structure modeling using MODELLER. Curr. Protoc. Bioinformatics. 2016; 54:5.6.1–5.6.37. 27322406 10.1002/cpbi.3 PMC5031415

[52] Ahvazi B., Coulombe R., Delarge M., Vedadi M., Zhang L., Meighen E., Vrielink A. Crystal structure of the NADP+-dependent aldehyde dehydrogenase from Vibrio harveyi: structural implications for cofactor specificity and affinity. Biochem. J. 2000; 349:853–861. 10903148 PMC1221214

[53] Lorentzen E., Hensel R., Knura T., Ahmed H., Pohl E. Structural basis of allosteric regulation and substrate specificity of the non-phosphorylating glyceraldehyde 3-phosphate dehydrogenase from thermoproteus tenax. J. Mol. Biol. 2004; 341:815–828. 15288789 10.1016/j.jmb.2004.05.032

[54] Gruez A., Roig-Zamboni V., Grisel S., Salomoni A., Valencia C., Campanacci V., Tegoni M., Cambillau C. Crystal structure and kinetics identify Escherichia coli YdcW gene product as a medium-chain aldehyde dehydrogenase. J. Mol. Biol. 2004; 343:29–41. 15381418 10.1016/j.jmb.2004.08.030

[55] Johansson K., El-Ahmad M., Ramaswamy S., Hjelmqvist L., Jörnvall H., Eklund H. Structure of betaine aldehyde dehydrogenase at 2.1 A resolution. Protein Sci. Publ. Protein Soc. 1998; 7:2106–2117. 10.1002/pro.5560071007 PMC2143847 9792097

[56] Perez-Miller S.J., Hurley T.D. Coenzyme isomerization is integral to catalysis in Aldehyde dehydrogenase. Biochemistry (Mosc.). 2003; 42:7100–7109. 10.1021/bi034182w 12795606

[57] Mizuguchi K., Deane C.M., Blundell T.L., Overington J.P. HOMSTRAD: a database of protein structure alignments for homologous families. Protein Sci. 1998; 7:2469–2471. 9828015 10.1002/pro.5560071126 PMC2143859

[58] Lee S., Blundell T.L. BIPA: a database for protein-nucleic acid interaction in 3D structures. Bioinformatics. 2009; 25:1559–1560. 19357098 10.1093/bioinformatics/btp243

[59] Ochoa-Montaño B., Mohan N., Blundell T.L. CHOPIN: a web resource for the structural and functional proteome of Mycobacterium tuberculosis. Database (Oxford). 2015; 2015:bav026. 25833954 10.1093/database/bav026 PMC4381106

[60] Murzin A.G., Brenner S.E., Hubbard T., Chothia C. SCOP: a structural classification of proteins database for the investigation of sequences and structures. J. Mol. Biol. 1995; 247:536–540. 7723011 10.1006/jmbi.1995.0159

[61] Sillitoe I., Lewis T.E., Cuff A., Das S., Ashford P., Dawson N.L., Furnham N., Laskowski R.A., Lee D., Lees J.G. CATH: comprehensive structural and functional annotations for genome sequences. Nucleic Acids Res. 2015; 43:D376–D381. 25348408 10.1093/nar/gku947 PMC4384018

