# Supplementary material for: XSuLT: a web server for structural annotation and representation of sequence-structure alignments
Source: Nucleic Acids Res. 2017 May 16;45(Web Server issue):W381–7. doi: 10.1093/nar/gkx421 (PMC5793734; doi:10.1093/nar/gkx421)
Supplement: Supplementary Data [file gkx421_supp.docx]

**Supplementary Materials**

**The XTEML Alignment Format**

XSuLT generates two forms of output: an XML containing the raw data of the alignment and annotations, with the extension .xteml, and a formatted HTML file, generated by the transformation of the XML file by an XSLT template, which is freely available under the MIT license at <https://github.com/blundell-lab/xsult>. The XML follows the basic format below. Elements in square brackets are optional, or conditional on sequence type; the | symbol delimits allowed options.

<alignment name="alignment id" numseq="total number of sequences" numstruc="number of sequences with structures">

For each sequence in the alignment:

<sequence id="id of sequence" length="ungapped length of sequence" type="structure|sequence|extraseq" [model="true" if sequence is a predicted model] [target="true" if sequence is meant to be the target for prediction] [pdbid="pdb code"]>

For each position in the aligned sequence:

<pos num="alignment position" resid="1-letter residue id" [resn="PDB residue number"] [ch="PDB chain"]>

For structure-type sequences:

<secstr>H(elix)|(b)E(ta)|C(oil)|P(ositive phi)</secstr>

[<solvac/> if solvent accessible]

[<hb_mc_co/> if hydrogen bond to mainchain carbonyl]

[<hb_mc_co/> if hydrogen bond to mainchain amide]

[<disulf/> if disulphide]

[<depth>depth value</depth>]

[<rinacc>rinacc value</rinacc>]

[<DSSP>full DSSP secondary structure assignment code</DSSP>]

[<targ_ident/> if template is identical to target]

[<interface>chain id of interacting chain</interface>]

[<ligbind resn="PDB residue number" ch="PDB residue chain"]>

ligand name</ligbind>]

[<contacts type="simple" dist="distance threshold">

<pos num="number of position in contact"/></contacts>]

For sequence-type sequences:

[<sspred conf="prediction confidence (range 0-9)">H|E</sspred>

if secondary prediction present]

[<disopred/> if predicted as disordered]

</pos>

</sequence>

For alignment level annotations:

<annotation>

For each non-gap position:

<pos num="non-gap alignment position">

<entropy>entropy value (range 0.0-1.0)</entropy>

<rmsd>rmsd value (in Ångstrom)</rmsd>

</pos>

</annotation>

</alignment>
